# Determination of Methotrexate in Biological Fluids and a Parenteral Injection Using Terbium-Sensitized Method

**Published:** 2011

**Authors:** Abolghasem Jouyban, Masoomeh Shaghaghi, Jamshid L. Manzoori, Jafar Soleymani, Jalil JalilVaez-Gharamaleki

**Affiliations:** a *Pharmaceutical Analysis Laboratory, Drug Applied Research Center, Tabriz University of Medical Sciences, Tabriz, Iran.*; b *Department of Analytical Chemistry, Faculty of Chemistry, University of Tabriz, Tabriz, Iran.*; c *Kimia Research Institute, Tabriz, Iran.*; d *Hematology-Oncology Research Center, Tabriz University of Medical Sciences, Tabriz, Iran.*

**Keywords:** Terbium-sensitized, 1, 10-Phenanthroline, Methotrexate, Injection solution, Urine, Serum

## Abstract

A new sensitive, simple and rapid method for determination of methotrexate (MTX) was developed based on quenching effects of MTX on the fluorescence intensity of Tb^3+^-1,10-phenanthroline complex. The fluorescence intensity was measured with excitation wavelength of 300 nm and emission wavelength of 545 nm and the quenched fluorescence intensity is proportional to the concentration of MTX in Tris-HCl buffer solution with a pH of 7.0. The effects of pH, time, order of addition of the reagents, temperature and the concentrations of Tb^3+^, buffer and 1,10-phenanthroline were investigated and optimized. The obtained linear range for the determination of MTX was 0.02-10 μg/mL. The detection limits (signal: noise = 3) was 0.015 μg/mL and the relative standard deviation for replicated determinations of 1 μg/mL of MTX was 1.9%. The proposed method is a simple, practical and relatively free from interference effects and was successfully applied to assess MTX in urine, serum and samples of an injection solution.

## Introduction

**Figure 1:**
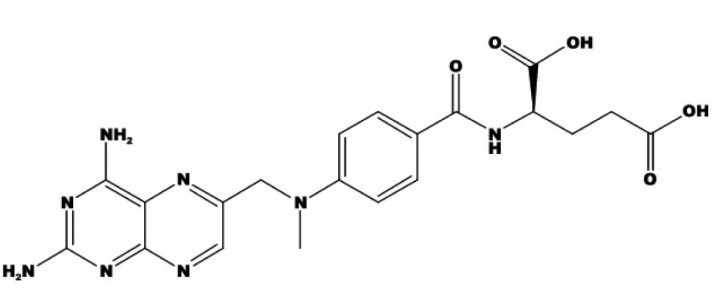
Structure of methotrexate

Methotrexate (MTX) or (2S)-2-[[4-[(2,4-diaminopteridin-6-yl) methyl-methylamino] benzoyl] amino] pentanedioic acid, (Figure 1) is an antineoplastic which acts as an antimetabolite of folic acid. MTX is a tight-binding inhibitor of dihydrofolatereductase, the enzyme responsible for the regeneration of tetrahydrofolate (THF) from dihydrofolate. Inhibition of this enzyme induces cellular depletion of THF cofactors, including 5-methyl-THF, and thereby blocks several folate-related metabolic processes. These include synthesis of purines and salvage of homocysteine to methionine (1-3). MTX is used in low doses (7.5-25 mg/week) in the treatment of certain connective tissues diseases such as rheumatoid arthritis, lupus and scleroderma (2-5). MTX is also used in high doses (> 1 g/m^2^ body surface) as an anti-cancer drug for treatment of certain forms of cancer (leukemia) (3, 6, 7). The efficacy of antifolate drugs is related to the extent of intra-cellular polyglutamation. In most cells, polyglutamation does not occur until the cell is exposed to 10^-6^ mol/L of MTX for at least 6 h (8, 9).

MTX is generally administered orally and after absorption, most of drug is excreted unaltered in urine. Less than 10% of MTX dose is converted to 7-hydroxymethotrexate (7-OH-MTX) in the liver. Both MTX and 7-OH-MTX are primarily excreted in urine although a small portion is also excreted in the bile. MTX binds to proteins (35-50%), while 7-OH-MTX possesses 91-95% albumin-binding and high tissue distribution. The usual terminal serum half-life of MTX is approximately 7-10 h (4, 10, 11). MTX concentration in plasma and other biological fluids is determined to investigate its pharmacokinetics and also to predict and prevent its toxicity when administered in high doses via intra-venous infusion (8, 12). Therefore, the establishment of highly sensitive and simple methods for the determination of MTX is of great importance in the area of pharmaceutical sciences.

Several methods for determination of MTX in biological fluids have been reported including fluorescence polarization immunoassay (13, 14), enzymatic assay (15), enzyme-multiplied immunoassay (16), radioimmunoassay (17), HPLC methods (18-21), capillary zone electrophoresis (8, 22), fluorimetric methods (23, 24) and voltammetry (25) methods. Fluorimetric methods are based on the oxidation of MTX to pteridine. Carboxylic acid was applied to measure the plasma levels of MTX in cancer patients. Most of these methods exhibit higher sensitivities, but their selectivities are usually unsatisfactory. Although immunological methods are the most commonly used ones, they are time consuming, need great care and also more skilled analyst. Chromatographic methods have the advantage of separating with the minimum interference from enzymes but involve set up cost, a complex extraction, purification procedure, longer analysis time, requiring relatively large volumes (> 0.1 mL) and lack of sensitivity. The major problem of electroanalytical techniques is their poor sensitivity. These methods, however are rapid and sensitive, possess inherent difficulties that limit their applications.

Considering above mentioned limitations and in order to improve measurement method of low concentration of MTX in biological samples, a simple, rapid and sensitive spectrofluorimetric method based on quenching of terbium sensitized fluorescence (TSF) is proposed in this work. Lanthanides have found applications in different fields of biomolecular and medical research. When the rare-earth ions (in particular Tb^3+^ and Eu^3+^) are chelated with ligand, their fluorescence can be dramatically enhanced. These chelates have unique fluorescence characteristics such as narrow spectral width, long fluorescence lifetime and large stokes shift. Therefore, terbium (ІІІ) chelates are often used as a fluorescence probe for determination of some substances, because of the high fluorescence quantum efficiency, based on the intensity fluorescence quenching or the enhancement of these chelates (26-29). Results of a previous study (30) demonstrated that Tb^3+^-1,10-phenanthroline (phen) complex has a strong intrinsic fluorescence of Tb^3+^. It was also found that its fluorescence could be quenched by MTX; therefore, a novel spectrofluorimetric method for determination of MTX could be developed.

## Experimental

*Reagents and solutions*

All chemicals and solvents were of analytical reagent grade. Double distilled water was used throughout this work. A 10^−2^ mol/L terbium (III) solution was prepared by dissolving theappropriate amount of terbium (III) chloride hexahydrate (TbCl_3_.6H_2_O) (Acros Organics, USA) in double distilled water and stored in polyethylene containers to avoid memory effects of terbium adsorbed on glass vessels.

MTX with a purity of more than 98% was purchased from Sigma. A 10000 μg/mL stock standard solution was prepared by dissolving 100.0 mg of MTX powder in approximately 2 mL of 0.1 mol/L sodium hydroxide and diluted to the mark in a 10 mL volumetric flask with 10% w/v NaCl solution. The stock solution was stored at -20°C. Appropriate MTX solutions of different concentrations (10, 100 and 1000 μg/mL) were prepared by dilution with distilled water, stored at 4°C and protected against light.

A 10^-2^ mol/L stock solution of 1,10-phenanthroline (Phen) (Fluka, Switzerland) was prepared in 10 mL ethanol and diluted to the mark in a 100 mL volumetric flask with double distilled water.

A 0.05 mol/L tris-(hydroxymethyl) aminomethane-hydrochloric acid (Tris- HCl) buffer solution was prepared by dissolving a desired amount of Tris-base (Merck, Germany) in 90 mL of water, adjusting the pH to 7.0 with HCl and making up the volume to 100 mL with water. Working standard solutions were prepared daily by successive dilution of the stock standard with water. MTX (50 mg/5 mL) injections were purchased from a local pharmacy store with the trademark of Ebetrex.

*Apparatus*

Fluorescence spectra and intensity measurements were performed using a Jasco FP-750 spectrofluotimetry (Japan) equipped with a 150 W xenon lamp, using 1.0 cm quartz cells. All measurements were performed at 25 ± 0.1°C using a Peltierthermostatted cell holder (Jasco, Japan). The pH of solutions was measured with Metrohm model 744 pH meter (Switzerland).

*Experimental procedure*

For the determination of MTX, the analytical procedure used to construct the calibration graph was as follow: 1 mL of 1×10^-3^ mol/L Tb^3+^ solution, 1 mL of 0.05 mol/L Tris-HCl buffer (pH 7.0) solution, 0.25 mL of 1×10^-3^ mol/L Phen solution and aliquots of working MTX solutions (10 and 100 μg/mL) were added to 5 mL volumetric flasks. The mixture was then diluted to the mark with distilled water. The final MTX concentration was in the range of 0.02-10 μg/mL. The solutions were thermostatted at 25 ± 0.1°C and the fluorescence intensities of the solutions and blank were measured at 545 nm using an excitation wavelength of 300 nm immediately after the addition of reagents (during 2 min). Both emission and excitation slits were set at 10 nm. The quenched fluorescence intensity of Tb^3+^-Phen by MTX was represented as I_0_/I_f_ in which I_f_ and I_0_ were the intensities of the systems with and without MTX, respectively.

*Sample preparation*

The proposed procedure for the determination of MTX was applied to a parenteral formulation (50 mg/5 mL, Ebetrex®, Unterach, Austria). In addition, MTX in human urine and serum was determined. For determination of MTX in an injection solution, the diluted samples were used.

*Experimental procedure for the determination of MTX in serum sample*

The analytical procedure used to construct the calibration graph was as follow: an aliquot of 100 μL serum with convenient amounts of MTX stock solutions (1000 and 10000 μg/mL) was added to 1 mL micro tubes. Then, samples were vortexed for 30 sec and after 15 min, 400 μL acetonitrile was added to remove proteins. Deproteinization was carried out by vortexing for 5 min and centrifugating for 15 min at 10000 rpm. Afterward, 0.5 mL of the supernatant was pipetted to a 10 mL volumetric flask and 0.5 mL was used to analyze as explained for the standard solution of MTX. MTX concentrations in final solutions after the dilution were in the range of 0.05-8.3 μg/mL. The fluorescence intensity of solutions and free drug serum (blank) was measured at 545 nm using an excitation wavelength of 300 nm. The recovery assay was carried out using the same procedure by addition of known amounts of MTX. Real serum samples taken from patients were also determined.

*Experimental procedure for the determination of MTX in human urine sample*

The analytical procedure used to construct the calibration graph was as follow: an aliquot of 50 μL urine with convenient amounts of MTX stock solutions (100, 1000 and 10000 μg/mL) was spiked and diluted to 10 mL with distilled water. Then, an aliquot 0.1 mL of the diluted samples in a 5 mL volumetric flask was measured as explained for standard MTX (1000 fold). The MTX concentration in final solutions after diluting is in range of 0.02-10 μg/mL. The fluorescence intensity of solutions and free drug (blank) urine was measured at 545 nm using an excitation wavelength of 300 nm. The recovery assay was carried out using the same procedure by addition of known amounts of MTX.

## Results and Discussion

*Fluorescence spectra*

**Figure 2:**
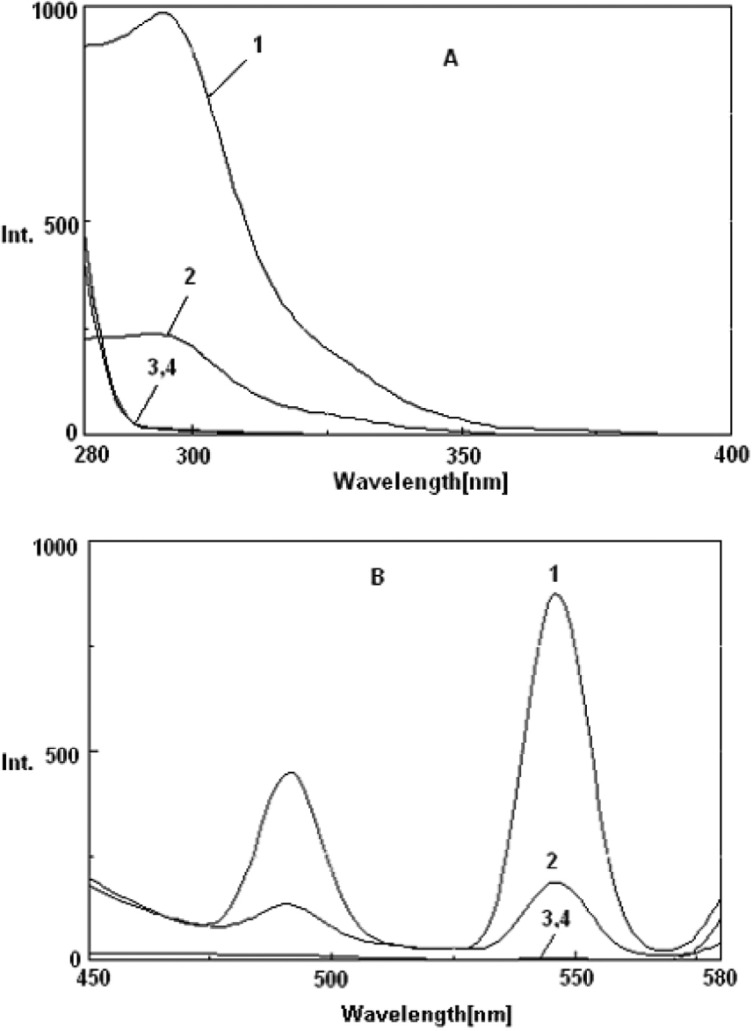
Terbium-sensitized fluorescence excitation (λ_em_= 545 nm) (A) and emission (λ_ex_= 300 nm) (B) spectra: (1) Tb^3+^-phen; (2) Tb^3+^-phen -MTX; (3) Tb-MTX; (4) Tb^3^. Conditions: [Tb^3+^] = 2×10^-4^ mol/L, [MTX] = 2 μg/mL, [phen] = 10^-4^ M, (Tris-HCl = 0.01 M, pH = 7.0).

Fluorescence excitation and emission spectra of (1) Tb^3+^-Phen, (2) Tb^3+^-Phen-MTX, (3) Tb^3+^-MTX and (4) Tb^3+^ systems are shown in Figure 2. The characteristic peak of Tb^3+^-Phen was observed with two emission peaks at 545 nm and 490 nm. The fluorescence spectrum of Tb^3+^-Phen-MTX system was similar to that of Tb^3+^-Phen; however, the fluorescence intensity was decreased by MTX. These results indicated that there was an interaction between MTX and Tb3+-Phen. Therefore, the Tb^3+^-Phen-MTX system was utilized in the assays of MTX. Since the quenched fluorescence intensity at 545 nm was higher, this wavelength was chosen to detect the fluorescence intensities throughout all the experiments.

*Factors affecting the fluorescence intensity of the system*

*Effect of pH and concentration of buffer*

**Figure 3:**
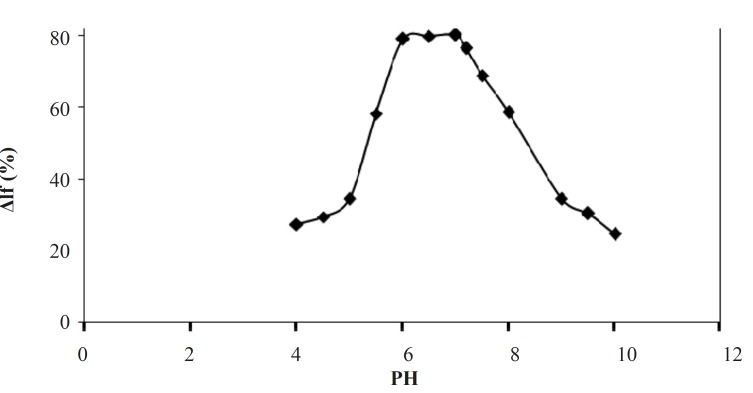
Effect of pH on the quenched fluorescence intensity Tb^3+^-phen -MTX system. Conditions: [phen], 2 ×10^-4^ mol/L; [Tb^3+^], 10^-4^ mol/L; [MTX], 2 μg/mL.ΔI_f_(%) = (I_0_–I_f_)/I_0 _× 100 in which I_f_ and I_0_ were the intensities of the systems with and without MTX, respectively

The effect of pH on the quenched fluorescence intensity (ΔI_f_(%)) of the Tb^3+^-Phen-MTX system in the pH range of 4.5-10.0 (Figure 3) was investigated. As it is obvious in Figure 3, the decreased fluorescence intensity (ΔI_f_(%)) of Tb^3+^–Phen complex is strongly dependent on pH and is reached to a maximum value between pH 6.8 and 7.2. Thus, pH of 7.0 was selected as the optimum pH.

This pH was adjusted by Tris buffer solution. Experiments indicated that the chemical nature of the buffer has also considerable effects on the quenched fluorescence intensity. The results showed that 1 mL of Tris-HCl (0.05 mol/L, pH = 7.0) in a final 5 mL was the most suitable concentration. Tris buffer also increases the obtained signal, probably due to its penetrating the coordination sphere of the chelate, giving rise to a synergistic effect.

*Effect of time and temperature*

**Figure 4:**
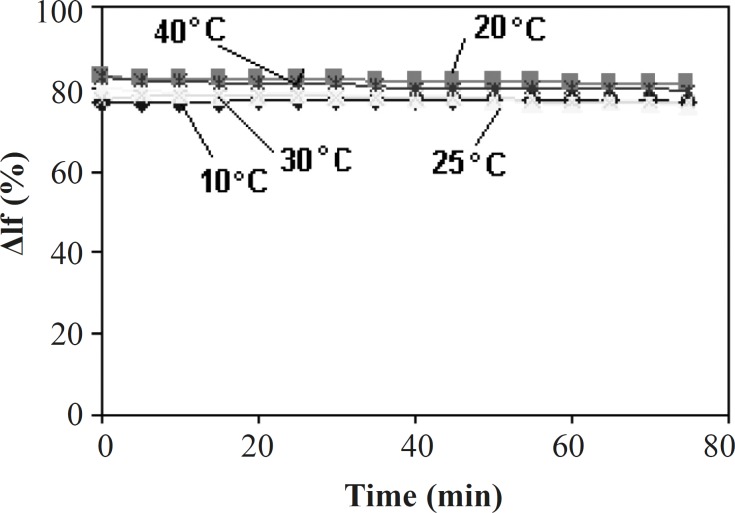
Effect of time on the fluorescence intensity and stability of Tb-phen-MTX system in different temperature. Conditions: [phen], 2 ×10^-4^ mol/L; [Tb^3+^], 10^-4^ mol/L; [MTX], 2 μg/mL; (Tris-HCl = 0.01 M, pH = 7.0).

Effect of time and different temperatures on the fluorescence intensity (ΔI_f_(%)) of Tb^3+^-Phen and Tb^3+^-Phen-MTX systems was studied within 80 min (Figure 4). As shown in Figure 4, in all temperatures, the fluorescence intensity of Tb3+-Phen and Tb3+-Phen-MTX systems is approximately constant. Therefore, the quenched fluorescence intensity of the systems is very stable and determination can be carried out immediately after the addition of all regents. The other results also showed that the quenched fluorescence intensity of the systems remain stable for more than 12 h.

*Effect of concentration of terbium*

**Figure 5:**
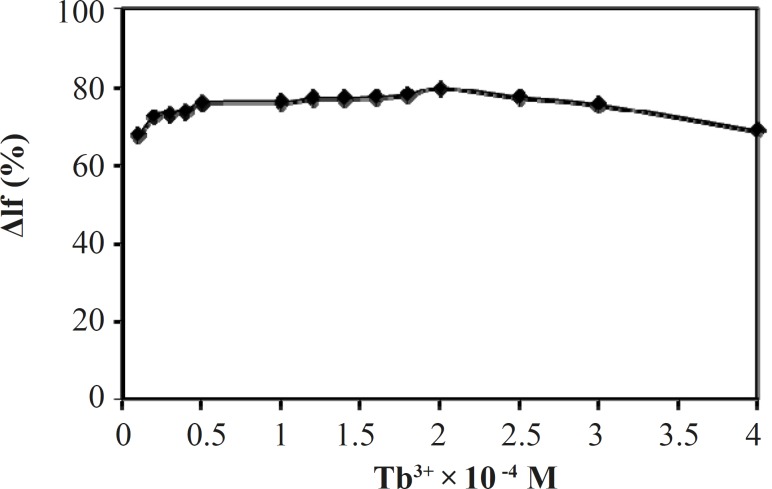
Effect of Tb^3+^ concentration on the quenched Fluorescence intensity. Conditions: [phen], 2 ×10^-4^ mol/L; [MTX], 2 μg/mL, (Tris-HCl, 0.01 mol/L, pH = 7.0).

The effect of the concentration of Tb^3+ ^on the quenched fluorescence intensity (ΔI_f_(%)) of Tb^3+^-Phen-MTX system in constant concentration of Phen was studied and results are shown in Figure 5. It can be seen that ΔI_f_(%) has been the highest when the concentration of Tb^3+^ in the mixture was 2.0 × 10^-4^ mol/L. Therefore, the concentration of Tb^3+^ in the mixture was chosen at 2.0 × 10^-4^ mol/L for further research.

*Effect of concentration of phen*

**Figure 6:**
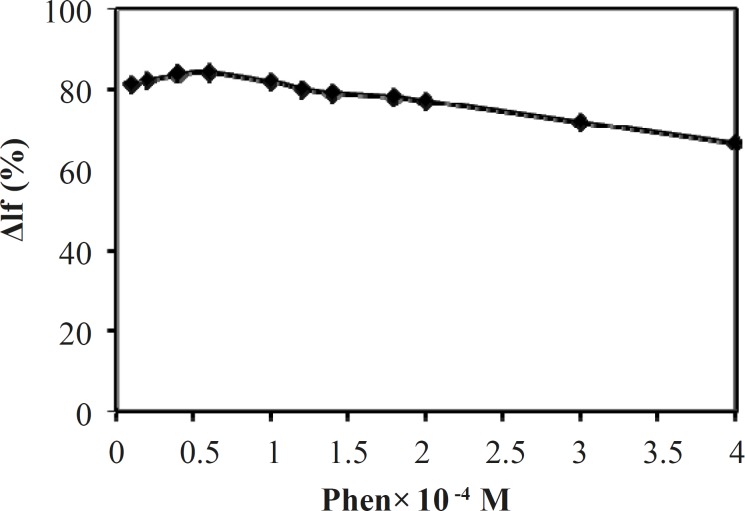
Effect of phen concentration on the quenched fluorescence intensity. Conditions: [Tb^3+^], 2 ×10^-4^ mol/L; [MTX], 2 μg/mL; (Tris-HCl, 0.01 mol/L, pH = 7.0)

The effect of Phen concentration on the fluorescence intensity (ΔI_f_(%)) of Tb^3+^-Phen-MTX system was studied and as the results show in Figure 6, it was found that ΔI_f_(%) of Tb^3+^-Phen-MTX system reached to a maximum value when the concentration of Phen was 0.5 ×10^-4^ mol/L. Therefore, this value was used for further study.

*Effect of addition order*

Finally, the effect of addition order was tested. For this purpose, a series of solutions with different addition orders of reagents and their corresponding blank solutions were measured at λ_ex_/λ_em_= 300 nm/545 nm. Based on the results and considering the system stability along with the quenched fluorescence intensity enhancement, the order of Tb^3+^, Tris-HCl, Phen and MTX was the best and followed in further investigations.

*Concentration ranges and calibration graphs*

By using the optimized conditions described above, a spectrofluorimetric method was developed for the determination of MTX in the biological samples. The measurements are based on the Stern-Volmer equations (31, 32) which describes dynamic or collisional quenching requiring contact between the excited luminophore and the quencher Q, as follows:

I_0_/I_f_ = 1 + K_q_τ_0_ [Q]

In this equation, I_0_ and I_f_ are the fluorescence intensities in the absence and the presence of a quencher, respectively; K_q_is the quenching rate constant (Lmol^-1^s^-1^) and τ_0_ is the fluorescence lifetime(s) in the absence of the quencher. The equation shows that a plot of I_0_/I_f_ versus the quencher concentration should give a straight line usable for the determination of the quencher. A high value of K_q_ leads to a low limit of detection for the quencher. The data showed that there is linearity between the MTX concentration and the quenched fluorescence intensity of the Tb^3+^-Phen system.

The calibration graph (the plot of I_0_/I_f_ versus the MTX concentration) (n = 10) was found to be linear in the range of 0.02 to 10 μg/mL for MTX and its equation was as follows:

I_0_/I_f_ = 1.0969 C + 1.0176 (r = 0.999)

Where I_0_/I_f_ is the quenched fluorescence intensity of Tb^3+^-Phen by MTX and C is the concentration of MTX expressed in μg/mL. Standard deviations (SD) for the slope and intercept of the calibration graph were ± 0.0037 and ± 0.0025, respectively.

*The limit of detection (LOD) and the limit of quantification (LQD)*

In accordance to ICH definition (33), the limit of detection was calculated as 3S_b_/m (where S_b _is standard deviation of the blank and m is slope of the calibration graph). Using this formula, LOD was found to be 0.015 μg/mL. The limit of quantification was defined as 10S_b_/m and found as 0.052 μg/mL.

*Precision and accuracy*

In order to investigate the precision of the proposed method **(**repeatability), series of six solutions of 0.05, 1.0 and 4.0 μg/mL of MTX were measured on the same day. By applying the ICH definition (36), the relative standard deviation (RSD) for six analyses was 0.5, 1.9 and 0.4%, respectively. To assess the day-to-day precision (intermediate precision), repeated analyses of 1.0 and 4.0 μg/mL of MTX (six analyses) were performed over one month and interday RSD were 2.9 and 0.8%, respectively.

*Specificity and interference study*

For the possible analytical application of the proposed method, a systematic study of the effects of coexisting substances that was likely in biological samples on the reduced fluorescence intensity (ΔI_f_%) was carried out under the optimal conditions. The effects of these species were evaluated by the addition of increasing concentration of various species to a fixed amount of MTX (2 μg/mL), under the same experimental conditions, until variation greater than 10% in analytical intensity was achieved. The tolerance levels of various interferents (*e.g. *ions, amino acids, proteins and saccharides) are summarized in Table 1. Careful examination of Table 1 revealed that the most substances in low amounts were found to show small effects on the determination of MTX under permission of ± 10% relative error. In practice, through the dilution of urine samples up to 1000-fold and deproteinization of serum samples and with applying standard addition method, interference-free determination of MTX is possible. Hence, the selectivity achieved by the proposed method is good and it is possible to determine the MTX. Folic acid or folinic acid were co-administered with MTX and their interferences were also investigated and the tolerance ratios were 0.3 and 0.2, respectively.

**Table 1:** Effects of common interferents on the determination of 2 μg/mL MTX

**Coexisting substance**	**Ratio of Coexisting substance to MTX**	**ΔI**_F_ **%**
**K** ^+^ ** (Cl** ^-^ **)**	1:75	0.8
**Na** ^+^ **(Cl** ^-^ **)**	1:150	2.4
**Ca** ^2+^ **(Cl** ^-^ **)**	1:75	4.4
**Ba** ^2+^ **(Cl** ^-^ **)**	1:75	-1.1
**Al** ^3+^ **(Cl** ^-^ **)**	1:0.25	-7.8
**Cr** ^3+^ **(Cl** ^-^ **)**	1:0.02	-3.5
**Au** ^3+^ **(Cl** ^-^ **)**	1:0.20	4.5
**Zn** ^2+^ **(Cl** ^-^ **)**	1:0.4	2.6
**Mn** ^2+^ **(Cl** ^-^ **)**	1:0.4	3.9
**Ni** ^2+^ **(Cl** ^-^ **)**	1:0.2	-1.3
**Cu** ^2+^ **(Cl** ^-^ **)**	1:0.15	4.1
**Cd** ^2+^ **(Cl** ^-^ **)**	1:0.25	2.3
**Co** ^2+^ **(Cl** ^-^ **)**	1:0.2	0.9
**L-Alanine**	1:75	3.6
**L-Cysteine**	1:25	-5.0
**Tryptophane**	1:70	3.5
**Glycine**	1:25	2.7
**L-Leucine**	1:125	-5.4
**Tyrosine**	1:25	0.8
**Uric acid**	1:0.025	-1.9
**Sacarose**	1:12.5	2.6
**Glucose**	1:12.5	-2.9
**Bovine serum albumin**	1:0.20	-3.7
**Folic acid**	1:0.25	-3.8
**Folinic acid**	0.2:1	-4.5

*Analytical applications*

*Determination of MTX in an injection solution*

The developed method was applied to the determination of MTX in an injection solution and the results were shown in Table 2. One injection (containing 50 mg/5 mL) was directly diluted to 100 mL and then analyzed using the standard calibration method. The concentration of MTX in the injection solution was found as 51.5 mg/5 mL.

**Table 2:** Determination of MTX in urine samples

**Added (μg/mL)**	Found* (μg/mL)	**Recovery (%)**	**RSD (%)**
**25.0**	26.0 ± 0.4	104.8 ± 1.7	1.5
**40.0**	40.5 ± 0.4	101.2 ± 1.2	1.0
**100.0**	99.5 ± 4.1	98.4 ± 1.9	4.2
**200.0**	209.5 ± 0.7	104.7 ± 0.4	0.3
**300.0**	314.5 ± 8.0	104.7 ± 2.7	2.5

* Mean of five observations ± standard deviation

*Determination of MTX in urine samples*

To demonstrate the usefulness of the proposed method, several aliquots of MTX were added to urine. The standard addition method was used for MTX determinations due to the possible matrix effect from urine. For the assays of MTX in samples of urine, the fresh samples must be diluted appropriately within the linear ranges of determination (1000 fold). A portion of these sample solutions was analyzed through the developed method, using the standard calibration method. In these conditions, there is no interference in determination of MTX and this method can be utilized for direct determination of MTX after dilution.

The precision and accuracy of the method were investigated by adding different concentrations of MTX. Recoveries obtained for different concentrations of MTX spiked to urine samples were between 98.4% and 104.8% (Table 2) with the RSDs less than 3%. Inter day RSD values for concentrations of 200 and 40 μg/mL were 0.7% and 1.5%, respectively. Therefore, the proposed method was easy to perform for the direct determination of MTX without requiring any pretreatment step.

*Determination of MTX in patient serum samples*

The developed method was applied to the determination of MTX in human serum samples after a prior protein precipitation using acetonitrile with serum (acetonitrile ratio of 1 : 2). It should be noted that the dilution (1000 fold) of serum samples following the protein precipitation step is necessary. The dilution diminishes the high background fluorescence measured for blank serum sample.

The precision and accuracy of the method were measured by the added-found procedure. Spiked serum samples were prepared with addition of different concentrations of MTX, around therapeutic concentrations in human serum (50-300 μg/mL) and analyzed on different days for measuring inter-day precision (using the calibration graph on each day). Intra-day precision was obtained by replicate analysesof the serum samples on the same day. The data of Table 3 demonstrate an acceptable precision and accuracy over the investigated concentration range. Recoveries obtained for different concentrations of MTX in spiked serum samples were between 97.5% and 110.5% (Table 3) with the RSDs less than 5%. Therefore, the method is appropriate for determination of MTX in serum and meets the requirement of rapidity for routine use in high dose MTX therapy. Also, the advantage of a low-cost and easily handling instrument should be taken into consideration.

**Table 3:** Recovery of MTX in serum samples

	**Added (μg/mL)**	Found * (μg/mL)	**Recovery (%)**	**RSD (%)**
**Intra-day**	50.0	55.2 ± 1.8	110.5 ± 3.3	3.3
	100.0	106.3 ± 2.6	106.7 ± 2.6	2.4
	250.0	254.5 ± 0.7	98.2 ± 0.30	0.30
	300.0	314.5 ± 4.0	104.9 ± 1.3	1.3
**Inter-day**	50.0	53.2 ± 2.4	106.4 ± 4.8	4.5
	100.0	106.4 ± 1.4	106.4 ± 1.4	1.3
	250.0	243.7 ± 3.7	97.5 ± 1.5	1.5
	300.0	323.0 ± 7.1	107.7 ± 2.4	2.2

*Mean of five observations ± standard deviation

The regression equation (n = 21) was I_0_/I_f _= 0.7918 C + 0.9953 (r = 0.999) and the standard deviations (SD) for the slope and intercept of the calibration graph were ± 0.0012 and ± 0.0078, respectively. Results of analysis of a real serum sample showed that MTX level was 2244 mg/L (sample was diluted up to 4000 times to be in the linear range of the calibration graph).

## Conclusion

A new sensitive method for simple and rapid determination of MTX was developed for the determination of trace amounts of MTX. Phen forms a binary complex with Tb^3+^, which emits at 545 nm. MTX can remarkably reduce its fluorescence intensity at this wavelength. The reduction in the fluorescence intensity of the Tb^3+^-Phen probe is proportional to the concentration of MTX. This method was very simple, precise and rapid and also was easily applied to the determination of MTX in real samples with good reproducibility and allowed the interference-free determination of MTX in real samples. The presented method is not very selective in comparison with the HPLC methods, but it is fast and requires small volume of serum or urine samples for determination of MTX and besides, no sample pre-treatment is required.
